# Cardio oncology: Digital innovations, precision medicine and health equity

**DOI:** 10.3389/fcvm.2022.951551

**Published:** 2022-11-03

**Authors:** Diego Sadler, Tochukwu Okwuosa, A. J. Teske, Avirup Guha, Patrick Collier, Rohit Moudgil, Abdullah Sarkar, Sherry-Ann Brown

**Affiliations:** ^1^Cardio Oncology Section, Department of Cardiovascular Medicine, Heart Vascular and Thoracic Institute, Cleveland Clinic Florida, Weston, FL, United States; ^2^Division of Cardiology, Department of Medicine, Rush University Medical Center, Chicago, IL, United States; ^3^Division of Heart and Lungs, Department of Cardiology, University Medical Center Utrecht, Utrecht University, Utrecht, Netherlands; ^4^Division of Cardiology, Department of Medicine, Medical College of Georgia at Augusta University, Augusta, GA, United States; ^5^Cleveland Clinic, Cardio Oncology, Department of Cardiovascular Medicine, Heart, Vascular and Thoracic Institute, Cleveland, OH, United States; ^6^Division of Cardiology, Department of Medicine, Medical College of Wisconsin, Milwaukee, WI, United States

**Keywords:** cardio-oncology, digital technology, precision medicine, artificial intelligence (AI), health equity (MeSH), health disparities

## Abstract

The rapid emergence of cardio-oncology has resulted in a rapid growth of cardio-oncology programs, dedicated professional societies sections and committees, and multiple collaborative networks that emerged to amplify the access to care in this new subspecialty. However, most existing data, position statements and guidelines are limited by the lack of availability of large clinical trials to support these recommendations. Furthermore, there are significant challenges regarding proper access to cardio-oncology care and treatment, particularly in marginalized and minority populations. The emergence and evolution of personalized medicine, artificial intelligence (AI), and machine learning in medicine and in cardio-oncology provides an opportunity for a more targeted, personalized approach to cardiovascular complications of cancer treatment. The proper implementation of these new modalities may facilitate a more equitable approach to adequate and universal access to cardio-oncology care, improve health related outcomes, and enable health care systems to eliminate the digital divide. This article reviews and analyzes the current status on these important issues.

## Introduction

There are currently 17 million cancer survivors in the United States, and it is anticipated that this number will grow to over 22 million by 2030 (1). Moreover, cardiovascular disease (CVD) is the leading cause of non-cancer morbidity and mortality in most cancer survivors (2), and it has been established that cancer patients have a 2–6 times higher CVD mortality risk than the general population, and that due to more effective cancer treatments and declining cancer-related mortality, CVD care is a critical component for these patients overall outcomes (2, 3).

Common risk factors for cancer and CVD, healthcare disparities, and an aging population, may lead to poor CVD outcomes for cancer patients (4). To meet the needs of this growing population, cardio-oncology programs have emerged, but they remain mostly confined to larger institutions, which are often large academic referral centers (5). However, a majority of cancer patients are treated at local/community settings without cardio-oncology programs, and therefore, expanding cardio-oncology knowledge to these practice settings may favorably impact the number of patients that will benefit from specialized care. The American College of Cardiology (ACC)'s National Cardio-Oncology Survey identified specific barriers to the implementation of cardio-oncology programs, including lack of infrastructure, funding, dedicated training programs, and lack of cardio-oncology guidelines (6).

Local cardio oncology programs can grow and succeed by utilizing available resources within their professional societies which can guide them, support their growth and facilitate global collaborations. For instance, the Florida American College of Cardiology (FCACC) and the Florida American Society of Clinical Oncology (FLASCO) assessed the needs of cardiologists and oncologists with a survey that revealed a remarkable lack of knowledge, awareness and cooperation between cardiologists and oncologists, and these findings led to an online education program for both specialties designed to mitigate these deficiencies (7, 8). Subsequently, members from 19 ACC state chapters, 6 ASCO chapters, and 9 International Cardio Oncology Society (ICOS) chapters coalesced in a collaborative network that assessed the impact of the COVID-19 pandemic in the cardio oncology community throughout the world (9). Later, this collaborative network became the foundation for the first global cardio oncology registry (G-COR), an international consortium designed to assess regional and international patterns of treatment, clinical and socioeconomic barriers, and their impact on outcomes in cardio-oncology care throughout the world (10). This and other collaborations reflect the growth in cardio-oncology throughout the world (11–13) and are examples of how collaborative academic and community practice based initiatives may improve access to quality cardio-oncology care.

However, these emerging collaborations and networks will have to overcome challenges in data collection and will be better equipped to address these challenges by using novel technologies like telemedicine and wearable devices, and by the adoption of precision medicine, machine learning, and artificial intelligence (AI). Precision medicine is a form of medicine that uses information about an individual's own genes or proteins to prevent, diagnose or treat disease. AI is the application of computers that mimic human cognition, and are capable of learning, thinking and making decisions or taking actions at much higher speeds than humans are capable of. It includes the application of machine learning, a form of AI that uses neural networks and deep learning with the utilization of a statistical technique that learns by training models with data. AI has been applied in medicine for various purposes including cancer detection and staging. AI and the use of precision, individualized/personalized medicine, present novel tools and pathways in cardio-oncology (14, 15). AI and precision medicine are efficient systems that streamline the identification and monitoring of cardio-oncology patients and may facilitate early recognition of cancer therapy related cardiotoxicities.

Cardiovascular imaging is garnering interest as a vital component in screening, surveillance and individualized management of cancer related CVD (16). Indices of clinical significance in cardiac imaging are left ventricular ejection fraction and global longitudinal strain derived from echocardiography (16, 17). There is growing interest into the application of AI to enhance accuracy, bandwidth and precision of cardiac imaging to maximize surveillance of cardiac dysfunction. In this scenario, large cardiac imaging data sets are integrated into interpretive algorithms through machine learning capable of predicting risk of CVD among cardio-oncology patients (16). Machine learning employs computer algorithms capable of processing information by evaluating and surmising patterns from large data sets using advanced statistical models.

The most common medical specialties among clinical trials conducted with AI are Oncology (26.3%) and Cardiology (15%) (18). As a medical specialty that bridges both disciplines, cardio-oncology could gain insight into social determinants influencing cardiovascular outcomes in cancer through population-based epigenomics, environmentomics, or populomics, and data in precision medicine. Such data and insight would help to provide individualized care (19) with a goal of reduced disparities.

In cardio-oncology, racial and ethnic minorities, particularly African Americans have higher incidence of cancer therapy related cardiotoxicity compared to Caucasians (20, 21). Historically individuals of low socioeconomic status, ethnic/racial minorities, and residents of rural areas tend to experience a “digital divide” in access and also in inclusion of data used to feed these technologies (21, 22). If existing healthcare bias and inequality remain unaddressed, they may easily be transferred and propagated by AI powered machine learning systems as they rely on imputation of existing data sets. The implication is inequity in resource allocation culminating in the disenfranchisement of patients from lower social economic status and racial/ethnic minorities compared to their Caucasian counterparts (23, 24).

In this article, we will review the utilization of artificial intelligence, machine learning, and precision medicine to improve cardio-oncology care, and the need for proper use of these modalities to reduce disparities in access to care for vulnerable populations.

## Precision medicine

**Figure 1:**
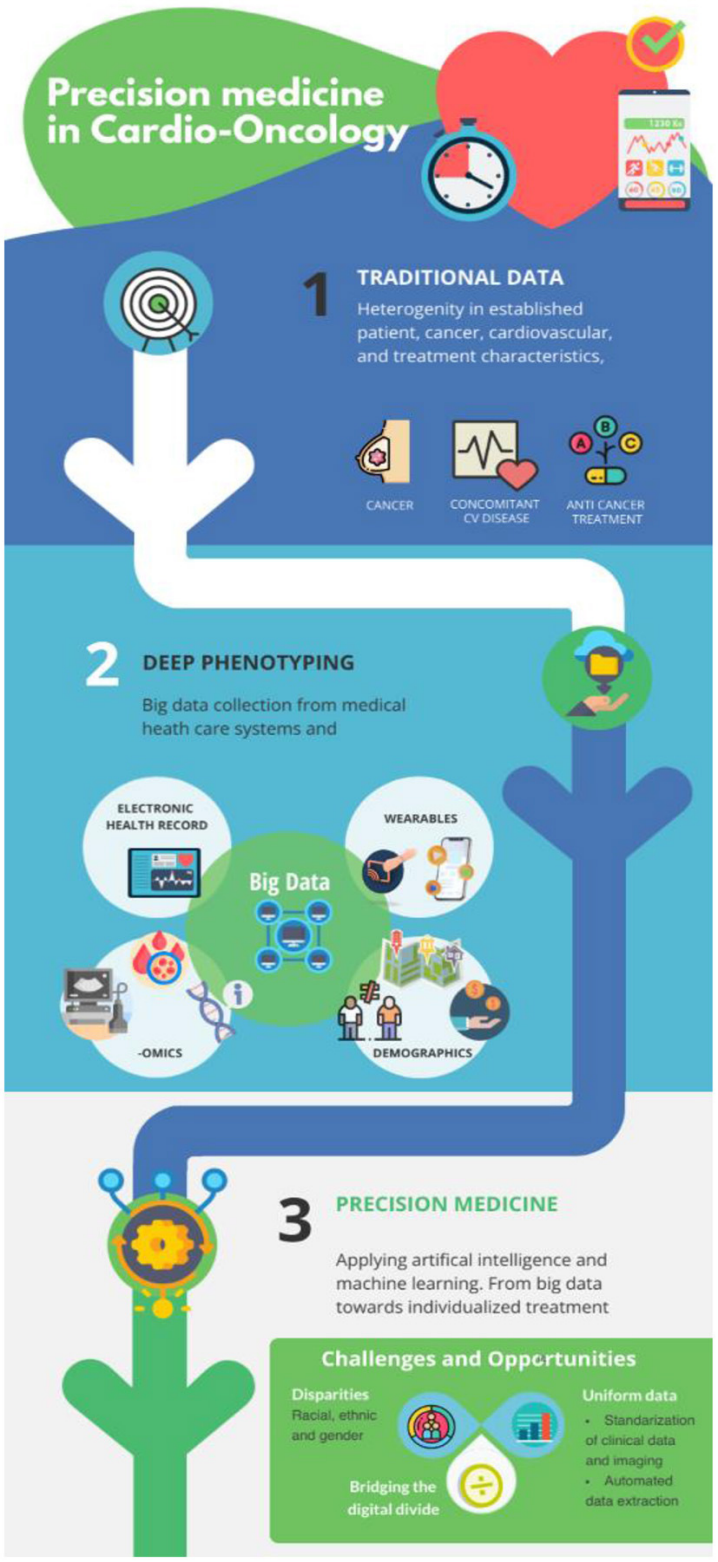
From traditional approaches toward precision medicine in cardio-oncology: Implementation of non-routine collected data will generate additional valuable information. AI and machine learning are instrumental for meaningful deep phenotyping of the cardio-oncology patient and lead to more personalized and better CV outcomes.

Precision medicine is a form of medicine that uses information about an individual's own genes or proteins to prevent, diagnose or treat disease. The precision medicine model is a medical model that proposes healthcare customization such that medical decisions, treatments, prevention strategies, practices, or products are being tailored to one or more subgroup of patients, rather than a one-drug-fits-all model (25, 26) (Figure 1). The model for precision medicine incorporates individual genetic, environmental, and experiential variability to name a few (25).

Precision oncology, defined as molecular profiling of tumors to identify targetable alterations, is fast developing and has entered the mainstream of oncology clinical practice (27). Leveraging burgeoning precision medicine platforms, precision oncology has revolutionized patient care and knowledge regarding the roles of genomics and the immune system in cancer and has therefore enabled the development of therapies targeted to specific molecular alterations or other biologic characteristics, including those implicated in immune suppression (28). Individual precision medicine platforms such as Tempus^®^, Genomoncology^®^, Missionbio^®^, have been utilized to identify genetic susceptibility to specific cancer treatments, which have in turn significantly improved survivorship for many cancer types (28). Tempus is the largest platform that organizes genomic profiling, digital pathology and AI. It is well-established in oncology, and has developed a cardiology platform. The GenomOncology precision oncology platform provides access to biomarkers based clinical trials and analyses complex mutations and chromosomal markers. Missionbio supports researchers and clinicians to study single-cell biology and facilitates development and delivery of precision medicine.

Such success in cancer treatment and survivorship has led the American Society of Clinical Oncology to develop the CancerLinQ program to create a large and broad ranging data platform in which clinical and genomic data can be collected and analyzed both for clinical and research purposes (29). Both traditional and novel cancer treatments are associated with a myriad of CVD complications and therefore make short- and long-term cardiovascular issues clinically relevant. Similar to precision oncology platforms, cardiovascular specialists are well-positioned to use precision medicine to impact clinical research with the goal of providing more targeted care that would improve individual and population health (25). Indeed, the American Heart Association (AHA) has developed the institute for precision cardiovascular medicine (30) and the AHA precision medicine platform^®^.

With the emergence of both oncology and cardiovascular precision medicine platforms, precision cardio-oncology is a necessary concept which would consider the cardiovascular and cancer treatment risks of each patient, and is well-positioned to predict cardiotoxicities and offer precise cardio-oncological care (31).

A clinically relevant risk stratification can be achieved by incorporating larger amounts of data aiming toward precision medicine. We can characterize an individual's cardiovascular biology from genetics, pharmacogenomics, proteomics, and radiomics (and medical imaging in general) (32) and combine these variables with personal traits derived from machine learning to guide precision treatment of heart failure (33). Furthermore, novel genetic discoveries from candidate gene approach or by genome-wide association studies (GWAS) have identified genetic variants in patients susceptible to CTRCD (34). These findings would further aid in the individualized approach to the cancer patient.

To expand precision medicine clinical applications and utilization, data collection at a large scale is critical, and Electronic Health Records (EHR) and large scale registries are instruments that will help to achieve this goal.

EHRs are built to share information with other health care providers and organizations including laboratories, specialists, medical imaging facilities, pharmacies, emergency facilities, and school and workplace clinics. As such, the EHR is a platform for information sharing that may lead to both clinical and research collaborations.

On the other hand, patient registries make use of observational study methods to collect uniform clinical data to evaluate specified outcomes for a specific population and provide a wealth of information about patient characteristics, patterns of care, and outcomes (35). Overall, whether using a patient registry or EHR systems, the linking of data obtained from these sources combined with molecular and genomic features has provided an invaluable resource to help create data-precision models for disease understanding and to improve care. Such precision medicine tools are particularly relevant in our field of cardio-oncology with the goal of anticipating and managing cardiovascular issues related to cancer therapy. To develop targeted preventive strategies, using the precision medicine approach necessitates the identification of cancer patients at increased risk for CVD and CVD death using both traditional cardiovascular risk factors and risk assessment tools such as blood biomarkers and global longitudinal strain. Ultimately, collaborations among cardio-oncology specialists are needed to assist clinicians in identifying, managing and preventing CVD among cancer patients at highest risk of serious or fatal CVD. Within the existing infrastructure, utilizing the precision medicine model in these collaborations is the obvious solution for future risk identification, prevention, and management in cardio-oncology.

## Precision medicine, the European perspective

There is a substantial evidence gap in diagnosing and treating cardiovascular complications in cancer patients since typical oncology and hematology trials exclude patients with underlying CVD. Conversely, most large cardiology randomized trials exclude patients with active cancer. Since cancer and CVD often overlap, it is worthwhile to investigate how cancer treatment modulates cardiovascular conditions and how cancer therapy-related cardiovascular dysfunction (CTRCD) would be best treated. Furthermore, real-world clinical data derived from health care records would most likely capture cardiovascular risk and complications in oncology patients, more accurately depicting a real-life incidence rather than data extracted from the 5% of all cancer patients participating in clinical trials (36).

As we work toward reducing these knowledge gaps, the Cardio-Oncology Study Group of the Heart Failure Association and the Cardio-Oncology Council of the European Society of Cardiology have already published position papers on baseline risk stratification (37) and on monitoring cancer patients receiving potentially cardio toxic anticancer therapies (38, 39). The position paper on baseline risk prediction was geared toward multi parameter models in different cancer therapies and aimed at a more personalized approach to the cancer patient. It should be stressed that most utilized risk factors models were based on expert opinion and that these models have not been validated. Even though this is an important first step, the proposed cardio-oncology risk prediction models still require further refinement, as highlighted during the Cardiovascular Round Table workshop by the European Society of Cardiology in January 2020 (40).

Recently, the European Union launched the Europe's beating Cancer Plan with €4 billion funding to its member states to develop more robust health care systems and to build an international platform to optimize data, digitalization, and big data analytics (41). Indeed, the European health systems and databases are diverse and fragmented, and there is a lack of harmonization of data formats, processing, analysis, and data transfer, subsequently leading to incompatibilities and lost opportunities (42). To address these infrastructural gaps, several recommendations have been proposed to move this field forward, including the Information and Communication Technology Competence document (42). The European Health Research and Innovation Cloud (HRIC) has been launched to facilitate access and to ensure high-quality health care data supported by an ethical and legal framework that is compliant with the General Data Protection Regulation in the EU (43).

The EU has been coordinating collaborative research in its member states since 2007 (44) and the recently launched Horizon Europe Program includes the main policy forum on personalized medicine (the International Consortium on Personalized Medicine, ICPerMed), providing guidelines and additional support over the next decade aiming at advancing research and ensuring high-quality data for personalized medicine (45).

Cardio-oncology would benefit from large data analysis and further development in personalized medicine due to the heterogeneity in baseline patient risk factors, concomitant diseases, and choice of anti-cancer treatment. That is also the case in Europe with the backdrop of cultural diversity and the differences between countries regarding health care access, guidelines, and clinical practice.

In response to Europe‘s beating cancer plan, the European Society of Cardiology (ESC) and the European Cancer Organization (ECO) have joined forces to put a spotlight on the cardiovascular diseases in cancer patients and survivors, and on the cardiovascular adverse effects of anti-cancer treatment emphasizing the need for further developing cardio-oncology research and healthcare within this plan (46).

## Informatics, big data, and machine learning in cardio-oncology

Informatics is the ability to use current technology to transform data into knowledge used for clinical or research purposes. Around the turn of the millennium, large national registries such as the ACC's National Cardiovascular Data Registry (NCDR), Medicare limited datasets, and Healthcare Cost and Utilization Project (HCUP) became available for secondary data analysis for cardiovascular research (47–49). These registries would be classified as big data which is defined as datasets too complex to be dealt with traditional data processing software. Several critical insights from these registries have led to designing better and more pragmatic clinical trials, leading to improved care for patients with CVD (50). However, multi-morbidity was almost always excluded in clinical trials, which has led to cardio-oncology care extrapolated from the cardiology literature wherein cancer patients were often excluded (51, 52).

Multiple thought leaders, societies, and healthcare systems have developed informatics platforms to mine the data collected from real-world patients. These initiatives have already been present in nationalized healthcare systems like Taiwan and Denmark, where a sizeable national dataset of multiple disease states exists (53, 54). The idea has been to organize the various parts of the patient's data in silos that belong to the same organization. The clinical data is compiled with billing, tumor registry, genomics, and electronic medical records data. The infrastructure is often supported by external sources such as the ASCO's Cancer LINQ Discovery dataset to fill in the missing elements that this dataset collects (55, 56).

While these registries are operationalized to generate some high-quality, real-world data, specific datasets are already contributing good quality cardio-oncology studies to the field. The Surveillance, Epidemiology, and End Results (SEER)-Medicare, Food and Drug Administration's Adverse Events Registry (FAERS), Women's Health Initiative (WHI), and the Pathways Heart Study, have resulted in high impact articles contributing to the field of cardio-oncology (57–60). For example, in a recent study using SEER-Medicare, atrial fibrillation in women with breast cancer was linked with an increased stage of breast cancer in a fully adjusted model (57). In addition, it was also noted that atrial fibrillation after breast cancer diagnosis increased the risk of cardiovascular mortality but not cancer-specific mortality, thus providing cardio-oncology-specific knowledge that is actionable and worth testing in a clinical trial setting (57).

While big data and informatics have provide valuable information, machine-learning and the use of AI have been equally valuable. In a patient–patient, network-based, machine learning approach that assessed an array of clinical variables, Troponin-T and NT-pro-BNP were predictors of cardiovascular risk in cancer patients (14). This methodology enabled the identification of 4 unique patient clusters with distinct *de novo* risk of CTRCD and all-cause mortality. Based on this AI based study, evaluation of troponin and NT-pro-BNP may identify patients most ideal for referral to cardio-oncology units that may benefit from preventive strategies for CV risk reduction, although further independent validation and clinical trials are warranted. In a separate analysis involving a large-scale machine learning–based approach, echocardiographic variables were also found to be associated with multiple types of cardiovascular outcomes, including coronary artery disease, atrial fibrillation, heart failure, stroke and myocardial infarction, in addition to *de novo* CTRCD (61).

Application of AI algorithms in cardio-oncology will require critical work to standardize clinical and imaging data. Furthermore, external validation of machine learning–based approaches using independent cohorts are necessary before implementation in patient care can be universally applied (16).

## Socio economic, racial and demographic disparities in cardio-oncology: The digital divide

Disparities in cardio-oncology have been linked to race, ethnicity, gender, socioeconomic status, lack of health insurance, language and cultural barriers, systemic discrimination, and challenges in access to preventive and high-quality specialty care (62). Regardless of cancer staging, compared to other ethnicities and races, Caucasian patients have higher chances of early diagnosis, undergoing aggressive treatment, and survivorship (63–66). Hispanic and Latinos in the United States make up 30.1% of the uninsured US population, compared to Caucasians (67). They are unlikely to undergo screening, surveillance and management of cancer therapy related cardiotoxicities (67). African American women with breast cancer have been shown to have higher risk of cancer and cancer therapy related cardiotoxicity than their Caucasian peers. These poor outcomes are multifactorial, stemming from the earlier onset of CVD, barriers to preventive care and monitoring, healthcare system bias, and other social determinants of health (62). Individuals of lower socioeconomic status have poorer cardiovascular health outcomes due to limited access to health including preventive screening, financial constraints or lack of insurance coverage, and low literacy levels (68, 69). Access to cardio-oncologic care is also constrained due to shortage of centers, training programs, and geography. Many at-risk patients reside in small towns/cities with limited access to cardio-oncologists, who tend to domicile in larger cities (68, 69). These data gaps collectively constitute an important data set currently missing from the US healthcare system's database that need to be harnessed and incorporated into AI or machine learning system to ensure adequate representation and general applicability.

There is unprecedented advancement in the use of innovative health technologies including AI, mobile health, wearable devices, and telemedicine in cardio-oncology. Unfortunately, limited access lead to disparities in the availability and the use of health technologies among marginalized groups including racial and ethnic minorities. Furthermore, AI algorithms are typically trained on existing data, which historically have had explicit and implicit biases against certain populations. Consequently, marginalized groups are susceptible to harm from erroneous predictions and resource withholding (23, 70). Additionally, much of the available data on genetically determined metrics and pharmacogenetics variations that inform current practice, are predominantly obtained from studies that predominantly include whites, thus making the accuracy and applicability to other racial/ethnic populations unclear (71–74). The collective phenomenon of limited access, bias, and other related components of the disparities in the availability and use of health technologies is known as the “digital divide”.

Cardio-oncology telemedicine can potentially expand access to patients in remote, resource-limited areas, as can online resources and mobile technologies (70, 71). Therefore, it is paramount to engage proactive measures emphasizing distributive justice principles toward the development of health innovation systems. Precision cardio-oncology, if appropriately implemented, has the potential to reduce bias in care, as management is stratified at the individual level and not based on demography. Furthermore, according to initial findings, African Americans are proficient in using electronic health and mobile technologies and are likely to participate in mobile health programs (72, 73).

## Discussion

In order to go from traditional approaches to precision medicine, new non-routine clinical and non-clinical data will be needed (Figure 1): the implementation of precision medicine, AI, and machine learning will be critical for meaningful deep phenotyping of cardio-oncology patients (Table 1). This approach will hopefully lead toward improved CV outcomes. If properly implemented, precision cardio-oncology may become the proper tool to eliminate the digital divide with algorithms based on biological, genetic, and molecular parameters that can equally benefit diverse populations.

**Table 1:** Summary of sources of data collection, AI/precision medicine and digital divide.

**Topics**	**Resources (R) and gaps (G)**	**What's available (A)/What's missing (M)**
Need for expanded patient access	- Local, state, national, international networks. R. - Cardiologists and oncologist' collaboration. G. - Telemedicine, wearables. R.	- Advocacy, education, cardio oncology programs. A. - Increased community practices involvement. M.
Data collection of clinical information	- Electronic health records (EHR). R. - Prospective registries. R. - Large clinical trials in cardio oncology. G.	- Clinical, laboratory, imaging, and pharmacy data sharing for clinical and research collaborations. A. - Observational uniform data collection to evaluate outcomes. A.
Precision medicine	- Epigenomics, proteomics, populomics. R. - Pharmacogenomics, Environmentomics. R.	- Dedicated cancer platforms and cancer LinQ. A. - AHA: Institute for Precision CV medicine. A. - Broad utilization in CV medicine. M.
Big data	- American College of Cardiology NCDR. R. - Medicine dataset. R. - SEER. R. - Healthcare Cost and Utilization Project (HCUP). R. - European Health Research and Innovation Cloud. R.	- Complex datasets. A. - Use of technology to transform data into clinical and research knowledge. A. - Large prospective datasets. M.
AI/machine learning	- Computers AI simulate human intelligence at much higher speeds. R. - Monitoring risk of cancer treatment related cardiotoxicities. R. - Established clinical practice. G.	- ML identified cardiotoxicity predictors: troponin, pro BNP, atrial fibrillation, CAD, CHF, CVA. A. - AI algorithms in echocardiography and imaging for diagnosis, prognosis, and surveillance. A.
Digital divide and health care disparities	- Racial minorities higher incidence of cardiotoxicity. G. - Individuals of lower socioeconomic status have poorer CV outcomes. G. - At-risk patients in rural areas have limited access to cardio oncology. G.	- Socioeconomic and racial data gaps need to be incorporated into AI/machine learning to ensure adequate representation and equitable solutions. M.

AHA, American Heart Association; CV, cardiovascular; NCDR, National Cardiovascular Data Registry; AI, Artificial Intelligence; SEER, Surveillance, Epidemiology and End Results; ML, Machine learning; BNP, Beta natriuretic peptide; CAD, coronary artery disease; CHF, heart failure; CVA, cerebrovascular accident.

Proper utilization of high-quality datasets, existing datasets from nationalized health care systems, and established and emerging collaborations through networks and professional societies looking at real-world data, EHR data and registries, will expand the understanding of risk stratification and the assessment of cardiovascular care of cancer patients in diverse populations, well-beyond the narrow scope of selected patient populations that are eligible to participate in clinical trials.

To bridge the digital divide, AI and machine learning systems data should undergo constant re-training and continuous monitoring to eliminate healthcare bias and undue influence from social determinants of health. Proper utilization and programming of these novel tools will result in a more personalized approach and subsequently impact quality of life for all patients regardless of background, demographics, ethnicity, or socio-economic background.

Overarching measures to mitigate inequities in cardio-oncology, including those attributable to innovative health systems would require increasing access to digital health platforms, unbiased precision care, deconstructing healthcare bias, and instilling confidence. Mandating appropriate level of inclusion of marginalized populations in clinical research and a multidisciplinary management approach is also necessary. These measures will facilitate the implementation of precision care for innovative health systems utilizing accurate and representative data that foster advantageous outcomes for all. Bridging the digital divide in this way will require a team-based strategy with contributions from all stakeholders: patients and their families, healthcare providers, third-party payers, community leaders, government agencies, and innovative technology companies and corporations. Such a multifaceted approach would facilitate developing consumer-focused digital health systems that are interactive, intuitive, current, and culturally and socially competent.

## Author contributions

All authors listed have made a substantial, direct, and intellectual contribution to the work and approved it for publication.

## Funding

S-AB this publication was supported by the National Center for Advancing Translational Sciences, National Institutes of Health, through Grant Nos. UL1TR001436 and KL2TR001438. Its contents are solely the responsibility of the authors and do not necessarily represent the official views of the NIH. AG this publication was supported by the Cardio-Oncology Program, Georgia Cancer Center, Medical College of Georgia at Augusta University, Augusta, GA, USA and Division of Cardiology, Department of Medicine, Medical College of Georgia at Augusta University, Augusta, GA, USA. AG is supported by American Heart Association-Strategically Focused Research Network Grant in Disparities in Cardio-Oncology (Grant Nos. #847740 and #863620).

## Conflict of interest

The authors declare that the research was conducted in the absence of any commercial or financial relationships that could be construed as a potential conflict of interest.

## Publisher's note

All claims expressed in this article are solely those of the authors and do not necessarily represent those of their affiliated organizations, or those of the publisher, the editors and the reviewers. Any product that may be evaluated in this article, or claim that may be made by its manufacturer, is not guaranteed or endorsed by the publisher.
